# Testosterone, oxytocin and co-operation: A hypothesis for the origin and function of music

**DOI:** 10.3389/fpsyg.2023.1055827

**Published:** 2023-02-13

**Authors:** Hajime Fukui, Kumiko Toyoshima

**Affiliations:** ^1^Nara University of Education, Nara, Japan; ^2^Osaka Shoin Women’s University, Higashi-osaka, Ōsaka, Japan

**Keywords:** origin of music, testosterone, oxytocin, sociality, cooperation

## Abstract

Since the time of Darwin, theories have been proposed on the origin and functions of music; however, the subject remains enigmatic. The literature shows that music is closely related to important human behaviours and abilities, namely, cognition, emotion, reward and sociality (co-operation, entrainment, empathy and altruism). Notably, studies have deduced that these behaviours are closely related to testosterone (T) and oxytocin (OXT). The association of music with important human behaviours and neurochemicals is closely related to the understanding of reproductive and social behaviours being unclear. In this paper, we describe the endocrinological functions of human social and musical behaviour and demonstrate its relationship to T and OXT. We then hypothesised that the emergence of music is associated with behavioural adaptations and emerged as humans socialised to ensure survival. Moreover, the proximal factor in the emergence of music is behavioural control (social tolerance) through the regulation of T and OXT, and the ultimate factor is group survival through co-operation. The “survival value” of music has rarely been approached from the perspective of musical behavioural endocrinology. This paper provides a new perspective on the origin and functions of music.

## 1. Introduction

From Aristotle (c. 335 B.C.) to Darwin (1874) and Spencer (1868), scholars have studied the origin and functions of music. From approximately 1900 to 1950, these authors were joined by comparative musicologist Sachs (1943), amongst others. This research was stalled by the anti-evolutionary environment that emerged in cultural anthropology after World War II (Wallin et al., 2000) but re-emerged at the end of the 20th century (Brown and Wallin, 2000; McDermott and Hauser, 2005; Trainor, 2015). Although there are several preliterate cultures, no culture is without music and language. Music is essential to human behaviour; however, its existence is a mystery, and there is no consensus regarding its origin and functions. Broadly, claims regarding this subject are divided into two categories: evolutionary adaptiveness (survival value) and evolutionary by-product (Pinker, 1997). Theories have ascribed a survival value to music, citing its benefits for social bonding and group cohesion (Brown, 2000), sexual selection (Darwin, 1874; Miller, 2000) or caregiving (mother–child interactions; Trehub and Trainor, 1998). However, these hypotheses are inconclusive (Beccacece et al., 2021).

These theoretical perspectives are based on the theory of evolution; however, most implemented approaches are based on psychology and sociology. The evolution and survival value of music have also been investigated from a biological perspective (e.g., Beccacece et al., 2021). Studies have found that music is closely related to important human behaviours and abilities, such as cognition, emotion, rewards and sociality (co-operation, synchronisation, empathy and altruism). Notably, the aforementioned behaviours are deeply linked to testosterone (T) and oxytocin (OXT). Neuropeptides [OXT and arginine vasopressin (AVP)] and steroid hormones (T and oestrogen) play central roles in the social lives of animals and humans (McCall and Singer, 2012), affecting cognition, emotions and motivation, which influence behaviour (van Honk et al., 2015). In both sexes, T and OXT influence reproductive behaviour and are vital in aspects of social behaviour, such as affiliation, social cognition, aggression, emotion and stress and anxiety. Indeed, they play a key role in psychiatric disorders that impair social communication and interaction, such as autism spectrum disorders, major depressive disorder, schizophrenia and personality disorders (Feldman, 2012; Brüne, 2016; Crespi, 2016; Bradley and Woolley, 2017; Dai et al., 2017; Bakker-Huvenaars et al., 2020). Furthermore, these diseases are related to musical behaviour (musicality and therapy; Foubert et al., 2017; Gassner et al., 2022). Why music is associated with important human behaviours and neurochemicals closely related to reproductive and social behaviours remains unclear. Also unclear is why music works for individuals with mental disorders that have no radical cure.

First, the neurochemical exploration of music (musical behavioural endocrinology) is in its early stages (Chanda and Levitin, 2013; Koshimori, 2019). Traditionally, research on music and endocrinology (biochemistry) has been conducted in the music therapy field because music can reduce stress. Moreover, investigations of the mechanism have demonstrated that a decrease in cortisol (C), also called the stress hormone, is responsible for reductions in stress (Koshimori, 2019). Since then, research has mainly focused on applying music for stress regulation using C as an indicator, as well as research on immune-related substances (Koshimori, 2019). As a research field, endocrinological research has until recently been conducted exclusively in music therapy. With the rapid development of higher brain function research using fMRI and PET in the 1990s, music started to gain attention from the perspective of clarifying emotions and creativity. Moreover, research using dopamine—a neurotransmitter—as an indicator of the relationship between reward and pleasure induced by music was conducted (Ferreri et al., 2019). Subsequently, the target substance was expanded to include endorphins and serotonin, and in recent years, research has centred on OXT as an indicator of sociability (affiliation).

Under these circumstances, few studies have examined the relationship between T and music, which plays critical role in human behaviour. Additionally, discussions from the viewpoint of origins and evolution have been scant (Fukui, 2001). Recently, research has demonstrated that listening to and playing music affects T and OXT and that music is closely related to social behaviours, such as co-operation, empathy and altruism (Fukui and Toyoshima, 2014); moreover, music has been approached from an evolutionary perspective (Harvey, 2020). However, despite the attention, fewer experimental studies have been conducted on OXT than on T (e.g., Bowling et al., 2022), and the origin or evolution of music from the perspective of the relationship between the two substances has not been investigated. Studies have shown that T and OXT have opposite effects on sociality and aggression (Crespi, 2016). Therefore, studies that analyse the relationship between T and OXT substances are necessary to understand the function and evolution of music. An approach solely based on a single substance is insufficient.

In this paper, we examine the endocrinological and psychological functions of human social and musical behaviours; demonstrate their relationship with T and OXT; and hypothesise that the emergence of music is linked to behavioural adaptations, which emerged as humans became increasingly social to ensure survival.

## 2. Human social behaviour, T and OXT

Humans are unique amongst the species with a social structure. As humans evolved, they formed social groups of various sizes and morphologies, based on the co-operation of genetically unrelated individuals (Fehr and Fischbacher, 2003). Cooperative behaviour has been observed in humans from the earliest stages of development (Tomasello, 2014; Grossmann, 2018), but its evolution remains unclear. Despite research on cooperative behaviour and altruism, their underlying molecular mechanisms and genetic basis remain unclear (Kalinowski et al., 2021).

However, the neuro-endocrinological study of social behaviour has been progressing (Soares et al., 2010; Trumble et al., 2015). Notably, hormones can serve as underlying mechanisms that influence behaviour in a functional manner. Understanding these proximate mechanisms might explain human psychology (Welling and Shackelford, 2019). Generally, hormones help in achieving specific behavioural goals in social contexts (Witczak et al., 2019). In both sexes, T and OXT influence reproductive behaviour and aspects of social behaviours such as co-operation (Feldman, 2012; McCall and Singer, 2012; Kurzban et al., 2015; Gangestad and Grebe, 2017; Gordon et al., 2017; Geniole and Carre, 2018; Servan et al., 2018).

Neuropeptides, such as OXT and arginine vasopressin (AVP) and steroid hormones T and oestrogen, play central roles in the social lives of animals and humans (van Anders et al., 2011; McCall and Singer, 2012). Neuropeptides and hormones affect cognition, emotions and motivation, all of which influence behaviour (McCall and Singer, 2012; van Honk et al., 2015). Endocrinological tradeoffs are required to maintain a balance between mating and rearing, the stability of the group between dominant and subordinate individuals, and simultaneous formation of multiple strong bonds between individuals (Witczak et al., 2019). T and OXT are involved in libido and nurturing and are key factors in understanding tradeoffs (Witczak et al., 2019). T is negatively related to sociality or tradeoffs, whereas OXT is positively related to sociality or tradeoffs (van Anders et al., 2011), with contradicting effects on the same cognition and behaviour (Holtfrerich et al., 2016). For example, the administration of T reduces the relation between the orbitofrontal cortex and amygdala and suppresses social behaviour, whereas OXT enhances the association with the amygdala and promotes social behaviour (Crespi, 2016). Furthermore, T and OXT interact (Gordon et al., 2017), having a notable effect on parental behaviour—maternal and paternal (Abraham and Feldman, 2018). Moreover, the interaction and increased rate between the two substances strongly correlate with behaviour (Jaeggi et al., 2015). Sex hormones such as T have organisational and activational effects on the human brain and can interact with the neurotransmitter system. These biological mechanisms can profoundly affect behaviour and the structural and functional regulation of the brain (Hornung et al., 2020).

T plays an important role in multiple physiological processes in the brain and can regulate the expression of specific genes by binding to the androgen receptor. Furthermore, T acts on neurotransmitter receptors and mediates non-genomic, neuro-active effects (Höfer et al., 2013). For example, T affects regulatory neurotransmitter systems in the brain, namely, key elements of dopaminergic and serotonergic neurotransmission (de Souza Silva et al., 2009). OXT exhibit strong functional binding with dopaminergic and opioid systems in the brain, the closely related neuropeptide hormone AVP and the steroid hormones T and oestrogen (van Anders et al., 2011).

As discussed in this section, certain aspects of T and OXT systems and their interaction have been documented. However, several questions remain (van Anders et al., 2011) regarding how these substances interact in the brain (Holtfrerich et al., 2018).

## 3. Musical behaviour as it relates to T and OXT

As the literature demonstrates, T and OXT are closely related to social cognition (Crespi, 2016). T is related to spatial abilities and memory (Vigil et al., 2016), although there are differences based on sex in its association with spatial–perceptual cognitive skills (Kimura, 2002; Voyer and Jansen, 2017). In women, high levels of T are associated with high skills in these areas, and in men, the opposite pattern has been observed (Gouchie and Kimura, 1991; Brosnan, 2006).

Musical ability, including composing, is related to the cognitive ability of spatial perception (Voyer and Jansen, 2017). Sex differences are also observed in musical ability studies; for example, females more easily recognise familiar melodies than males can (Miles et al., 2016). Musical perception and cognition are also influenced by T and OXT (Fukui and Toyoshima, 2008).

Hassler (1991) conducted the first investigation on the relation between musicality (composition) and T and deduced that they are strongly correlated and that this correlation differs based on sex (Hassler and Gupta, 1993). For male composers, lower T levels were associated with more highly-rated compositions, whereas amongst female composers, higher ratings were received by those with higher T values. T values are sex-dependent, but values are continuous, not discrete. There is an overlap in testosterone levels between men and women (Sudai, 2017). Following this line of evidence, Hassler (1991) concluded that an optimal level of T would promote creative musical behaviour, similar to the optimal level of T for cognitive function (Hogervorst et al., 2010; Holland et al., 2011). Because musical ability is a type of spatial–perceptual cognitive ability (Hassler, 1992), there is probably an optimal level between continuous T for men and women and may be at the bottom of normal male T range and at the top of normal female T range.

A weak-to-moderate but consistent association exists between OXT and creativity (e.g., novelty-seeking, extraversion, divergent thinking and problem solving; De Dreu et al., 2015). For vasopressin, a posterior pituitary hormone with a similar structure to that of OXT (they differ by two amino acids), an association between its receptor [arginine vasopressin receptor (AVPR)] and musical ability has been reported. The AVPR1A gene has one of the strongest links to musical activity and related behaviours, according to genome-wide linkage and association studies (Koshimori, 2019). In vertebrates (e.g., fish and birds), the vasopressin peptide signalling system is involved in musical signals (in review Ebstein et al., 2012), and an association between musicality and AVPR1A has been reported (Ukkola et al., 2009; Fukui and Toyoshima, 2013; Granot et al., 2013; Szyfter and Witt, 2020).

Research on the effects of music (both listening and playing) on T, OXT, and other substances is still in its infancy and will therefore take some time to elucidate.

To the best of our knowledge, the studies that have examined the relationship between T and OXT and music are listed in Table 1. According to this, at this time, there is only one study in which OXT was administered to humans and the relationship to music was examined. The study showed that when OXT was administered intranasally to performers, performance stress was alleviated. Although not in music, a study using the interpersonal finger-tapping paradigm showed that dyads administered OXT were more synchronised than dyads administered a placebo (Gebauer et al., 2016). However, there were no studies that administered T.

**Table 1:** Music, T and OXT.

	Year	Author	Methods	Results
T × Music	1991	Hassler (1991)	Subjects: adults and adolescents	Creative musical behaviour was associated with very low T in males and high T in females
	2001	Fukui (2001)	Subjects: 70 people (35 men and 35 women, mean age 21 years) Stimuli: subjects listened to various genres of music for 30 min. (1) favourite music, (2) Gregorian chant, (3) Mozart, (4) jazz, (5) popular music and (6) silence	When listening to their favourite music, T decreased in men and increased in women
				Sex differences are inversely correlated, as is the talent for music composition
				Positively correlated with the preference for the music heard, i.e., the more enjoyment of the music, the larger the fluctuation
				Male: favourite music↓, Gregorian chant↓, Mozart↓, jazz↓, popular music↓, silence unchanged
				Female: favourite music↑, Gregorian chant↑, Mozart↑, jazz↑, popular music↑, silence↓
	2003	Fukui and Yamashita (2003)	Subjects: 88 healthy college students (44 males and 44 females) Stimuli: (1) 30 min of listening to music, (2) 30 min of listening to music with visual stress (documentary film without sound, including violent scenes), (3) 30 min of visual stress without music and (4) 30 min of silence	Significant differences between the sexes in how music affected T
				Music decreased T in males, but increased T in females
				C decreased with music in both sex
				T decreased by 14% amongst male subjects and increased by 21% amongst female subjects
				Male: music↓, stress with music↑, stress↑, control↑
				Female: music↑, stress with music↑, stress↑, control↑
	2011	Fukui and Toyoshima (2011)	Subjects: 42 female volunteers were enrolled. Mean age was 72.9 years Stimuli: 90 min of choir activity, four sessions (once a month)	The group with low T rose after singing in a chorus; conversely, T values decreased in the group with high T
				Anxiety and sore index decreased (POMS)
				Cognitive test results (WAIS) memory task (Silverman and Eals’ Object Location Memory Task)
				Mental rotations test (Vandenberg and Kuse Mental Rotations test “3-dimensional”) also improved
				Low T:↑
				High T:↓
	2012	Fukui et al. (2012)	Subjects: 6 patients with an established diagnosis of Alzheimer disease (6 females, mean age 81.8 years) Stimuli: (1) talking by therapist, (2) singing by therapist and (3) talking and singing by therapist	17β-estradiol and T levels increased during the therapist’s singing and talking sessions
				T: Therapist only↑, Music only unchanged, Music therapy↑
	2013	Borniger et al. (2013)	Subjects: music student: 21 (12 males, 9 females) non-music student: 40 (15 males, 25 females)	Female music majors had higher T than non-majors; high-ranked students had higher T than low-ranked students
				Male subjects’ results were less powerful than females
	2013	Fukui and Toyoshima (2013)	Subjects: 21 subjects (10 males and 11 females, mean age 35 years) Stimuli: (1) preferred music (chill-inducing music) and (2) disliked music	Listened to their favourite music (chill-inducing music); T decreased in males and increased in females
				T levels declined in males when they listened to both types of music
				The 17-beta estradiol levels increased in males with both types of music, whereas the levels increased with chill-inducing music but declined with disliked music in females. Advanced Measures of Music Audiation
				(AMMA) scores were higher for the short repeat length-type AR than for the long repeat length-type
				Male: chill-induced music↓, disliked music↓
				Female: chill-induced music↑, disliked music↓
	2013	Jezova et al. (2013)	Subjects: 14 healthy volunteers 14 male volunteers (aged 21–29 years) Stimuli: listening to music played forward (pleasant) or backwards (unpleasant) Familiar and unfamiliar music	T, vasopressin and OXT levels were considerably higher when unknown sounds were present compared with when familiar music was playing
				Male: unfamiliar sounds↑
				Unfamiliar: −2.71 ± 10.32 ng/mL/90 min
				Familiar: −53.85 ± 19.26 ng/mL/90 min
	2015	Beck et al. (2015)	Subjects: intervention: 13 participants (2 men and 11 women, mean age 43 years) control: 7 participants (2 men and 5 women, mean age 48 years) Stimuli: guided imagery and music used in music therapy	No significant changes were found in T
	2022	Bowling et al. (2022)	Subjects: young adult choir (*n* = 71) Stimuli: vocal production mode (singing vs. speaking) and social context (together vs. alone)	T was not significantly different (measured in male subjects only)
				OXT decreased after each condition, but significantly greater decreases were observed for speaking than for singing
	2022	Chéour et al. (2022)	Subjects: Male patients with mild AD (*n* = 26) were divided into four groups. Control (Co) group: *n* = 6 Participated in physical rehabilitation (PR) group: *n* = 6 Music therapy (MT) group: *n* = 7 MT + PR group: *n* = 7	Salivary T levels increased and C levels decreased significantly in the PR, MT, and MT + PR groups compared with the Co group
				The increase in T was particularly pronounced in the MT + PR group compared with the other groups
				The MT group also showed a significant increase in T values compared with the PR group
				In the PR, MT and MT + PR groups, T level changes correlated positively with changes in MMSE and negatively with C levels
				T: Co↓, PR↑, MT↑ and MT + PR↑
OXT × Music	2009	Nilsson (2009)	Subjects: 40 patients with open-heart surgery randomly allocated to either music listening during bed rest (*n* = 20) or bed rest only (*n* = 20) Stimuli: control group (15 men and 5 women, mean age 67 years) and music group (17 men and 3 women, mean age 64 years)	OXT increased in the music group
				OXT
				Music group: post1 (immediately after) ↑ and post2 (30 min later) ↑
				Control group: post1 (immediately after)↓
				Pre-value vs. post-value 1 mean (range): music group +3·95 (−10 to 28) (pmol/L), control group −5·45 (−29 to 8) (pmol/L)
				Pre-value vs. post-value 2 mean (range): music group +5·90 (−22 to 22) (pmol/L), control group −3·90 (−43 to 19) (pmol/L) and post2 (30 min later)↑
	2014	Kreutz (2014)	Subjects: 21 participants (16 female, 5 male; age range: 18–65 years) were identical in both sessions Stimuli: singing condition and chatting condition	Subsequent comparisons of means reveal that OXT increased significantly after singing
				Singing: ↑ (before session mean 13.044 pg./mL, after session mean 18.083 pg./mL)
				Chatting: NS (before session mean 14.282 pg./mL, after session mean 15.898 pg./mL)
	2015	Keeler et al. (2015)	Subjects: 2 males and 2 females (jazz vocalists, university students) Conditions: standard performance (SP), improvised performance (IP)	ACTH concentrations decreased in both conditions but more significantly in the pre-composed singing condition
				Mean plasma OXT increased only in response to improvised singing, with no significant difference between improvised and pre-composed singing conditions
				Effects of pre-composed and improvised group singing on OXT are less clear
				OXT: SP condition↓, IP condition↑
	2016	Fancourt et al. (2016)	Subjects: total 193 cancer: carers (*n* = 72), bereaved. Carers (*n* = 66) and patients (*n* = 55). Stimuli: participate choir	Significant decrease in C, beta-endorphin and OXT
	2016	Gebauer et al. (2016)	Subjects: 98 females (non-musicians). Stimuli: OXT or placebo, tapping (four conditions)	OXT improves synchronisation to an unresponsive partner’s behaviour through a reduction in tapping variability
	2017	Schladt et al. (2017)	Subjects: 38 student chorists. Two cohorts: one with 21 participants (males: *n* = 9, females: *n* = 12) and one with 17 participants (males: *n* = 8, females: *n* = 9). Stimuli: solo singing and choir	OXT was significantly reduced after choir singing but did not change in response to solo singing
				OXT showed high intra-individual stability, whereas C fluctuated between days in the same participant
				OXT concentration decreased significantly after chorus but did not change in solo singing.
				OXT: Solo singing↑, Choir↓
	2017	Ooishi et al. (2017)	Subjects: 26 healthy men (mean age 29 years). Stimuli: slow-tempo music and fast-tempo music	OXT increased in the slow-tempo music sequence.
				C decreased after listening to the fast-tempo music sequence
	2017	Yuhi et al. (2017)	Subjects: 23 boys and 5 girls (aged 8–15 years). Stimuli: Taiko (Japanese drum) performance	OXT concentrations were increased to various degrees after the activity sessions
	2021	Dağli and Çelik (2022)	Subjects: 73 mothers with premature infants admitted to the neonatal intensive care unit (NICU). Stimuli: music intervention, oxytocin massage intervention and control	Mother’s milk production was substantially higher during the music session than other session, which was followed by oxytocin massage sessions
	2021	Eerola et al. (2021)	Subjects: 62 women (divided into a low characteristic empathy group and a high characteristic empathy group). Stimuli: sad music or nothing	When compared with the no music condition, the high empathy group’s PRL and OT levels were significantly lower after listening to music
				The high empathy group reported a more positive mood and higher ratings of being moved by the music than the low empathy group
				No significant changes in C and adrenocorticotropic hormone
	2021	Greenberg et al. (2021)	Review Article	
	2022	Bowling et al. (2022)	Subjects: young adult choir (*n* = 71). Stimuli: vocal production mode (singing vs. speaking) and social context (together vs. alone)	T was not significantly different (measured in male subjects only).
				OXT decreased after each condition; significantly greater decreases were observed for speaking than for singing
	2022	Cohen and Nuemann (2022)	Critique of Greenberg et al. (2021)	Greenberg et al. (2021) hypothesised that music increases oxytocin levels, reduces social isolation and promotes social connectedness
				However, Greenberg et al. did not cite previous studies accurately and their hypothesis remains uncertain
				According to previous research, considerable uncertainty remains concerning the oxytocin response to music, such as oxytocin levels being reduced or unchanged by music (group singing)
	2022	Osório et al. (2022)	Subjects: 54 male professional singers with different levels of musical performance anxiety (42% high)	OXT administration during the performance and immediately after stress resulted in significantly more positive ratings of the performance than under placebo conditions. In contrast, placebo conditions resulted in more negative ratings
			Stimuli: intranasal OXT (24 IU), placebo	OXT minimises social stress, particularly during performance
	2022	Palumbo et al. (2022)	Subjects: 30 adults with post-stroke hemiparesis	Oxytocin levels did not change significantly in either group
			Conditions: Music Upper Limb Therapy-Integrated (MULT-I), home exercise programme.	

As already mentioned, musical ability is a type of spatial perceptual–cognitive ability, and the relationship between spatial perceptual–cognitive ability and T values is the same as that between musical talent (musical creativity) and T values. T values and talent are also known to have a sex-dependent inverse relationship. In males, low T values indicate high talent, whilst in females, conversely, high T values indicate high talent. To begin with, T levels in men and women are sex-dependent, and in healthy adults, T levels are said to be 5%–10% (1/10–1/20) of those in men when compared with blood testosterone levels. As we have already noted, several researchers have argued that somewhere in the difference between male and female T levels lies the optimal value for spatial perceptual–cognitive ability and musical talent.

Second, a relatively large number of studies have examined the relationship between music and T in healthy subjects, and the results are consistent. That is, music listening affects T values, but there are gender differences there. Particularly, music listening decreases T levels in men and increases them in women. The existence of sex differences in musical talent is also very interesting and will be discussed again in the Hypothesis section.

Conversely, the studies that have examined the relationship between OXT and music have not produced consistent results, so we are not in a position to say anything definitive about the effects of music. However, it is certain that music (both listening and playing) does affect OXT values.

## 4. Music, brain areas, sociality, T and OXT

Music has been consistently shown to activate a reward pathway similar to that associated with primary (food, sex, addictive drugs, friends and loved ones) and secondary reinforcers (money; e.g., Salimpoor et al., 2013). Simultaneously, it is deeply associated with brain regions of pro-sociality (co-operation, empathy, altruism; Harvey, 2020; Greenberg et al., 2021). Regions involved in the pleasure and emotion evoked by musical stimuli overlap with sociality and compare the limbic system (hippocampus, parahippocampal gyrus, amygdala and cingulate cortex), ventral striatum (nucleus accumbens), superior temporal gyrus, caudate nucleus, insular cortex, thalamus, orbitofrontal cortex, prefrontal cortex, dorsal prefrontal cortex, dorsolateral prefrontal cortex, inferior prefrontal cortex and supplementary motor cortex (in review Ferreri et al., 2019; Harvey, 2020).

T and OXT play an important role in emotion and are involved in activity in the aforementioned areas. Studies have shown that T is associated with limbic system activation, including the amygdala (e.g., Höfer et al., 2013). T can directly affect orbitofrontal function by interacting with local androgen receptors (Diekhof and Kraft, 2017). The circulation of T activates the androgen receptor and is a source of oestrogen for the brain (Juntti et al., 2010). Moreover, OXT exerts its effects by interacting with dopamine and the mesolimbic neurotransmitter system (Xiao et al., 2017). Brain structures affected by T comprise the hippocampus, hypothalamus, frontal cortex and cerebellum (Pillerová et al., 2021). Many of these areas are closely linked to the neural activity of OXT (Møller, 2021). Androgen receptors are found in the limbic, mesolimbic system and related areas. OXT receptors (OTR), vasopressin receptor 1a (V1aR) and vasopressin receptor 1b (V1bR) are expressed throughout the auditory and mesolimbic pathways (Johnson and Young, 2017). The ventral striatum/nucleus accumbens are the most relevant neural regions to music pleasure (Mueller et al., 2015). These structures are connected with the dopaminergic reward pathways, and dopamine has been suggested as relevant to music’s reward value (Blood and Zatorre, 2001; Ferreri et al., 2019). Subsequent findings have supported this hypothesis (Ferreri et al., 2019). Moreover, the dopamine system is regulated by T (Alarcón et al., 2017) and OXT (Gamal-Eltrabily and Manzano-García, 2018).

## 5. T and OXT are involved in social vocalisation

Vocalisation is an essential means of communication for mammals, from mice to humans, conveying important information on an individual’s reproductive, social and emotional state; location and identity: and the presence of food, relatives and predators (Tschida et al., 2019). Vocalisation is also central to music. As many theories on the origin of music suggest, music and language may be descendants of earlier forms of oral communication that disappeared amongst ancestral hominids (Leongómez et al., 2022). Many vocalisations can facilitate social interactions by reducing the uncertainty of the intentions and possible actions of the signaller. Such interactions help establish and maintain social bonds, contributing to successful reproduction (Cheney and Seyfarth, 2018).

Sex hormones are related to vocal behaviour in various species, including humans (Puts et al., 2016). Sex steroid hormones modulate vocal behaviour and regulate processing at various levels of the ascending auditory pathway (Caras, 2013).

According to studies on newborn vocalisation (babbling) and articulatory abilities in male and female children aged 5 months, T levels are related to auditory–vocal learning. Five-month T concentrations were negatively correlated to articulatory skills in babbling (Quast et al., 2016). Furthermore, there are sex differences in the perception of emotional prosody. Women are better at recognising emotional prosody than men (Lambrecht et al., 2014), and T may play an important role in this process (Fujisawa and Shinohara, 2011).

Humans have sex hormone receptors in their vocal folds, including the larynx (Kirgezen et al., 2017), and their development is closely related to T levels (Markova et al., 2016). Moreover, sex differences have been observed in emotional prosody recognition abilities. Adolescent females were more sensitive to emotional prosody than males. This finding suggests that sex differences for emotional prosody recognition emerge in adolescence, during which T levels become higher in males than females (Fujisawa and Shinohara, 2011), and that T is negatively correlated with aspects of social vocalisation.

Similar to T, OXT is involved in vocal processing in many animals, such as fish (Huffman et al., 2012), mice (Marlin et al., 2015) and humans (Theofanopoulou et al., 2017). However, unlike T, OXT positively correlates with social vocalisations. In humans, OXT positively correlates with maternal vocalisations, and maternal OXT levels during pregnancy positively correlate with motherese (Feldman, 2012), an infant-directed vocalisation (IDV; Fernald, 1992). Exposure to the mother’s voice increases daughters’ OXT levels (Seltzer et al., 2010). OXT administration in fathers also increases vocal synchronisation with their offspring (Weisman et al., 2014). OXT enhances the speaker’s vocal expression and social communication (Spengler et al., 2017). Although motherese has been associated with origins of music and language (Trehub, 2001; Falk, 2004), it is characterised by the exaggeration of vocal intonation (Saint-Georges et al., 2013; Adriaans and Swingley, 2017) and is not a form of music *per se*.

Vocal intonation transmits emotional information in many animals (Filippi et al., 2017), and thus the combination of emotional prosody and vocal expression may be at the origin of music (Darwin, 1874; Brown, 2000; Juslin and Laukka, 2003; Panksepp, 2009; Snowdon and Teie, 2010; Brown, 2017). Zimmermann et al. stated that the components of affective prosody in human speech and music have phylogenetic roots in non-human mammalian speech communication systems (Zimmermann et al., 2013). In summary, T is negatively correlated with synchrony and prosody, and OXT is positively correlated with these factors. OXT leads to increased synchrony (Gebauer et al., 2016; Josef et al., 2019), an important feature of music (and language). Similarly, prosody (pitch) is synonymous with musical melody.

## 6. T and OXT coordinate cooperative behaviour

Homo sapien is an ultra-social animal (Tomasello, 2014) and engages in cooperative behaviour regardless of the social group’s size. This unique ability to cooperate may have enabled humans to become Earth’s dominant species (de Waal, 2014).

From a neurobiological perspective, human pro-sociality has been suggested to be deeply rooted in neuroendocrine structures, with hormones regulating cooperative behaviour (Soares et al., 2010). These hormones include T and OXT, key neuromodulators of human social behaviour (van Honk et al., 2016; Geniole and Carre, 2018; Liu et al., 2019; Marsh et al., 2021).

Scientific investigations (particularly those involving neural imaging and physiological and psychological findings) have demonstrated that music plays a role in promoting co-operation within groups of genetically unrelated humans (Kniffin et al., 2017), altruistic behaviour (Fukui and Toyoshima, 2014) and social bonding (Savage et al., 2020). T and OXT play a role in cooperative behaviour and may therefore modulate co-operation in humans. Although studies have highlighted an association between OXT and social behaviour (Savage et al., 2020), few have examined the relationship between music and co-operation or altruism using OXT as an indicator.

OXT promotes synchronisation in the mother–child, father–child and other relationships (Feldman, 2017), whereas T inhibits synchronisation (Gordon et al., 2017). Rhythmic synchronisation is associated with cooperative, collaborative and pro-social behaviours (Cirelli, 2018). Furthermore, interpersonal behaviour synchronisation in infants increases pro-social behaviour (Tunçgenç et al., 2015). Movements synchronised with music promote cooperative behaviour in groups (McNeill, 1995) and increase group cohesion and social co-operation (Ellamil et al., 2016) amongst children (Kirschner and Tomasello, 2009; Cirelli, 2018). Music promotes communication (Spiro and Himberg, 2016) and triggers pro-social behaviour (Aucouturier and Canonne, 2017). It promotes the development of cognitive and motor skills and increases affinity and pro-sociality (Aucouturier and Canonne, 2017). Group singing can also increase intimacy and promote social cohesion (Pearce et al., 2016).

Empathy is a mechanism that causes altruistic behaviour (de Waal, 2012) and a central mechanism stimulated by emotions evoked by music (Juslin and Västfjäll, 2008). Studies on music and empathy have demonstrated that listening to and playing music promotes empathy (e.g., Fukui and Toyoshima, 2014). Furthermore, studies have found that music increases trust in others and promotes altruistic behaviour (Fukui and Toyoshima, 2014). When empathy was evoked, OXT and T levels were assumed to increase and decrease, respectively. Concentrations of OT were substantially lower with music than without it in highly empathic individuals. In a self-reported survey, highly empathic individuals reported that music enhanced their positive mood and increased their perception of being moved to a greater extent than low empathetic individuals did (Eerola et al., 2021).

The scope of the relationship between music and altruism was also shown using the dictator game (DG), an experimental measure of altruism commonly used in social psychology and economics wherein one participant (the dictator) receives a donation and then allocates it amongst the other anonymous participants (the recipients). In that experiment, DG was conducted with an in group (IG) and an out-group (OG) to determine whether participants listening to their preferred music (chill-inducing) affected altruistic behaviour (Fukui and Toyoshima, 2014). After listening to their preferred music, “dictators” increased the amount of money allocated to the recipient regardless of whether the recipient was in the IG or OG. The results indicate that music might encourage altruistic behaviour beyond ethnocentrism.

## 7. Hypothesis

Humans are hyper-social animals with distinctive cooperative behaviour relative to other species (Tomasello, 2014; Henrich and Muthukrishna, 2021), a high degree of social tolerance and a significant capacity for engaging in interpersonal assistance and collaboration (Tomasello, 2009).

As for co-operation, Darwin was troubled, because it runs counter to the theory of natural selection: the struggle for survival and the struggle for mating. The ability to cooperate emerged before advances in human cognition and culture (Hayes and Sanford, 2014).

Cooperative behaviour evolved and developed during the Pliocene and Pleistocene eras (approximately 5–10 million years ago) in specimens that branched from a common ancestor with chimpanzees (Meindl et al., 2018). These individuals experienced severe environmental selection pressure in an arid, unstable ecosystem, which contributed to the development and selection of cooperative traits for survival (Jetz and Rubenstein, 2011). Individuals with these abilities were selected *via* the evolutionary process.

The background explanation for this behavioural change is that the environmental selection pressure required males to invest in females. Examples of these behaviours include cooperative breeding (reproduction; Hrdy and Burkart, 2020; i.e., offspring of a bred female are cared for by others in the flock) and cooperative foraging (Tomasello, 2020; the flock or group collaboratively obtains food and other resources). Enabling these two co-operation types required new capabilities, especially (1) social tolerance and suppression of reactive aggression to help and cooperate with others and (2) cooperative–communicative abilities to enable co-operation.

Cooperative–communicative abilities may have co-evolved in humans and the coevolutionary relationship between vocal communication and group-level co-operation is not unique to humans of the ape lineage, but likely existed in the last common ancestor with chimpanzees (Mine et al., 2022).

These behavioural traits may have been caused by two endocrinological characteristics: reduced androgen responsiveness (reduced levels of circulating testosterone or reduced density of androgen receptors or decreased sensitivity in adults) and increased oxytocin activity (Cieri et al., 2014; Hare, 2017).

As aforementioned, T is generally correlated with aggression; thus, a decrease in T tends to decrease aggression. T is also negatively correlated with tolerance (sociability). Conversely, OXT generally has the opposite effects of T, increasing tolerance (emotional contagion, empathy) towards relatives.

The reduction of androgen during the process of humanisation has also been identified at the genetic level (the deletion of the androgen receptor). These genes were involved in steroid hormone signalling and neural function, and such deletions may have promoted brain size and upright walking and strengthened pair bonding (Reno, 2017). Aggressive, antisocial males with high T values are not expected to invest as fathers (Lovejoy, 2009; Potts, 2013; Clark and Henneberg, 2015), because high T values are negatively associated with paternal care (Abraham and Feldman, 2018) and vocalisations (Weisman et al., 2014) and were thus excluded from the selection (Lovejoy, 2009).

Moreover, T and OXT enabled the acquisition of other capabilities (cognition and vocalisation). T correlates with cognitive spatial abilities, and there is an optimal level between continuous T for men and women; OXT is also related to cognition, especially enhancing emotional cognition; and T has a negative effect on communication skills, whereas OXT has a positive effect. Thus, the action of both substances probably confers a high degree of cooperative–communicative abilities to the genus Homo. As aforementioned, there is an optimal level of T for spatial–perceptual cognitive ability, including musicality, and a presumption is that once T levels enter the optimal range, cognitive function improves. OXT is also involved in social information processing and memory (Guastella and MacLeod, 2012) and affects the recognition and retention of socially relevant information (Kinsley et al., 2015).

Babbling and IDV, a communication form that connects parents and their offspring, is universal in human language and may be unique to humans (Oller et al., 2019; Mehr et al., 2020). Babbling is rich in prosody. In IDV, mothers greatly change vocal timbre when speaking to their infants (Piazza et al., 2017). Moreover, IDV can be used by fathers and alloparents (Saint-Georges et al., 2013). This type of prosodic communication was probably also used amongst adults. Notably, IDVs have the requirements of music (pitch and rhythm) and can be referred to as primitive music (proto-music). Prosody is repeated to ensure that the intent is conveyed (rhythm) and that the range of intonation is increased (frequency modulation: melody). In addition, strength and weakness are added. These (rhythm, melody and dynamics) are the building blocks of music.

T and OXT are deeply involved in human Babbling-IDV-ADV (adult-directed vocalisation)-Prosodic vocalisation, T and OXT have an antagonistic effect on prosody and IDV is negatively correlated with T but positively correlated with OXT levels. The decrease in T and relative increase in OXT that occurred during the evolutionary process may have resulted in a quantitative increase in IDV and qualitative refinement, giving rise to proto-music.

Tone-rich proto-music (and features of modern music) results from a coordination of T and OXT, enabling emotional inflexion, synchronisation and the expression of empathy, facilitating cooperative breeding and alloparenting (paternal care), both of which are specific to human society.

The response of T levels to music differs by sex: a decrease in men and an increase in women, interestingly, the same changes that occur during love, which the process of pair bonding in humans (Marazziti and Canale, 2004). The decrease in males is probably a promotion of co-operation by suppressing reactive aggression. In interpreting the increase in females, the steroid/peptide theory provides (van Anders et al., 2011) a clue. It divides aggression into two types: antagonistic and protective aggression, both of which are associated with increased T. However, the aggression type depends on fluctuations in oxytocin. Whether this finding can be generalised remains unclear. If music increases OXT, the increased T in women can be interpreted as a correlate of protective aggression to protect their children and mates.

The difference due to sex in this reaction is considered the result of an ancestral survival strategy. In males, pro-social behaviour (co-operation) and pair bonding decrease T and increase OXT, whereas in females, T and OXT increase pro-social behaviour and pair bonding (Marazziti and Canale, 2004).

**Figure 1:**
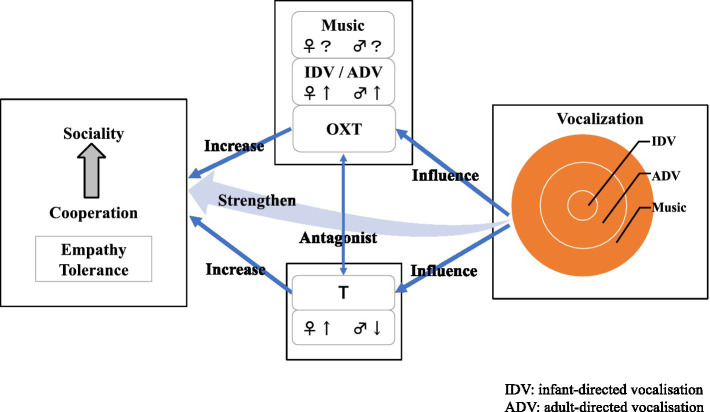
Relation of testosterone, oxytocin and co-operation.

In summary, the proximate factor in the emergence of music is in behavioural control (aggression and social tolerance) through regulation of T and OXT, and the ultimate factor is group survival through co-operation. Music promotes co-operation and altruism, but it also promotes co-operation and altruism between groups, from mother and child to pairing and beyond (Figure 1).

## 8. Limitations and future research

The literature on music and endocrinology is still in its infancy. Although, this manuscript is based on research in the field of musical behavioural endocrinology, its qualitative approach may limit its effects in literature. The paucity of biochemical studies, especially of OXT, may limit the strength of our observations. T and OXT alone may not provide a comprehensive understanding of the endocrinological mechanisms of music. Future studies using fMRI, which can directly visualise neuronal activity, would be a powerful way to examine the effects of music and steroids on brain function.

## Data availability statement

The original contributions presented in the study are included in the article/supplementary material, further inquiries can be directed to the corresponding author.

## Author contributions

HF conceived of the presented idea and developed the theory. KT investigate and verified the theory. All authors contributed to the article and approved the submitted version.

## Conflict of interest

The authors declare that the research was conducted in the absence of any commercial or financial relationships that could be construed as a potential conflict of interest.

## Publisher’s note

All claims expressed in this article are solely those of the authors and do not necessarily represent those of their affiliated organizations, or those of the publisher, the editors and the reviewers. Any product that may be evaluated in this article, or claim that may be made by its manufacturer, is not guaranteed or endorsed by the publisher.
